# Supplementary material for: Exploration of Nanosilver Calcium Alginate-Based Multifunctional Polymer Wafers for Wound Healing
Source: Pharmaceutics. 2023 Feb 1;15(2):483. doi: 10.3390/pharmaceutics15020483 (PMC9968014; doi:10.3390/pharmaceutics15020483)
Supplement: Supplementary file 1 [file pharmaceutics-15-00483-s001.zip › pharmaceutics-2183436-supplementary.pdf]

pharmaceutics

Article

Exploration of Nanosilver Calcium Alginate-Based Multifunctional Polymer Wafers for Wound Healing

Ernest Man [1], Claire Easdon [2], Iain McLellan [2], Humphrey H. P. Yiu [3] and Clare Hoskins [1,*]

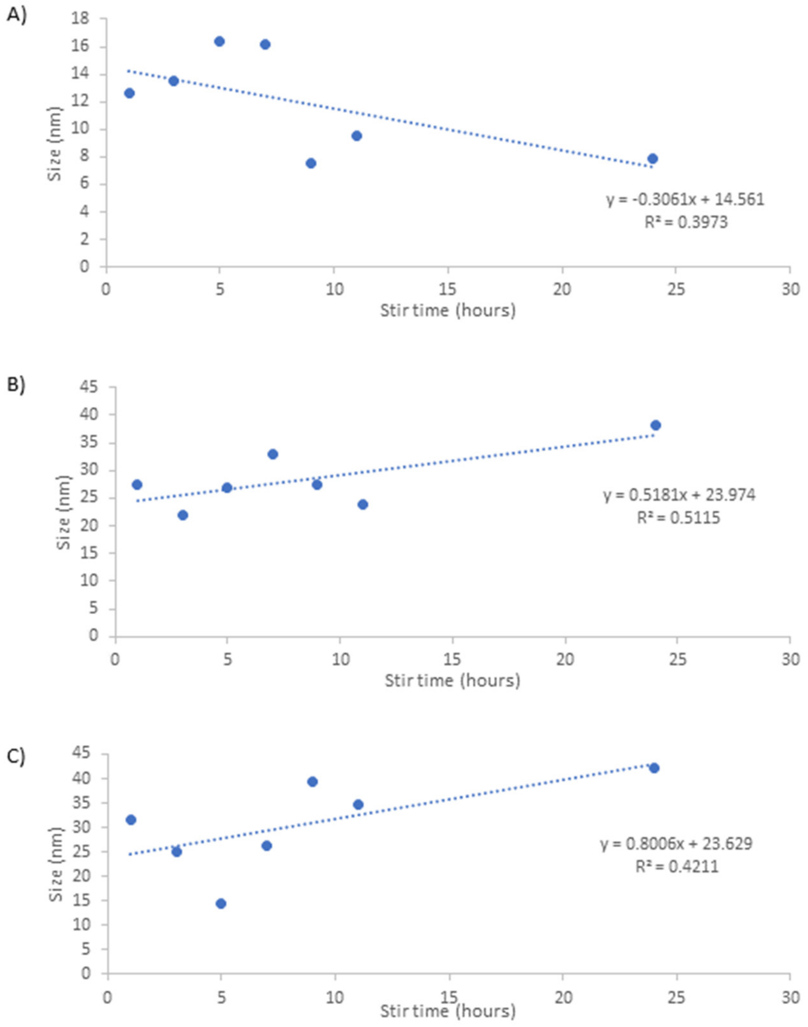

Figure S1 Changes in standard deviation for NAA samples. (**A**) 1%w/v, (**B**) 5%w/v and (**C**) 10%w/v

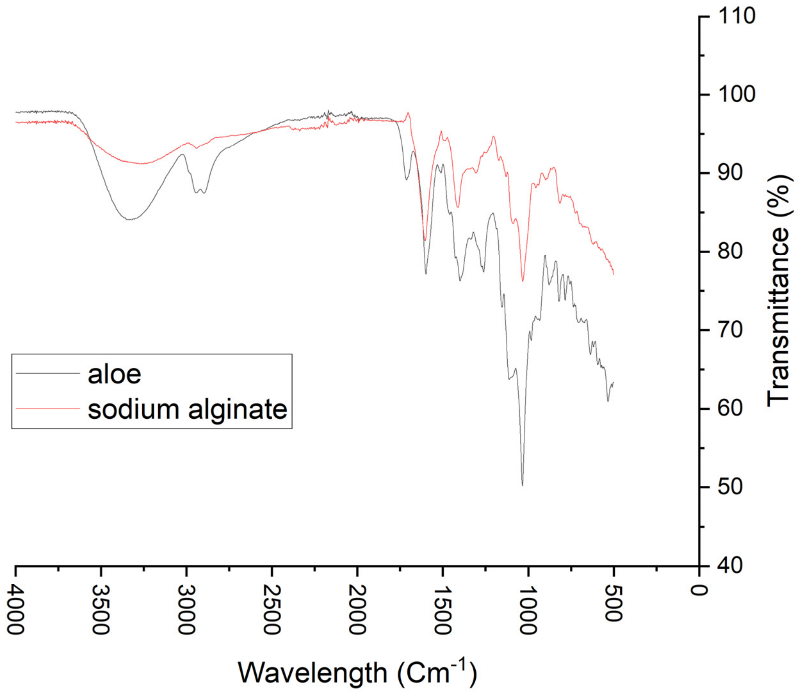

Figure S2 FTIR spectrum of pure aloe and pure sodium alginate.

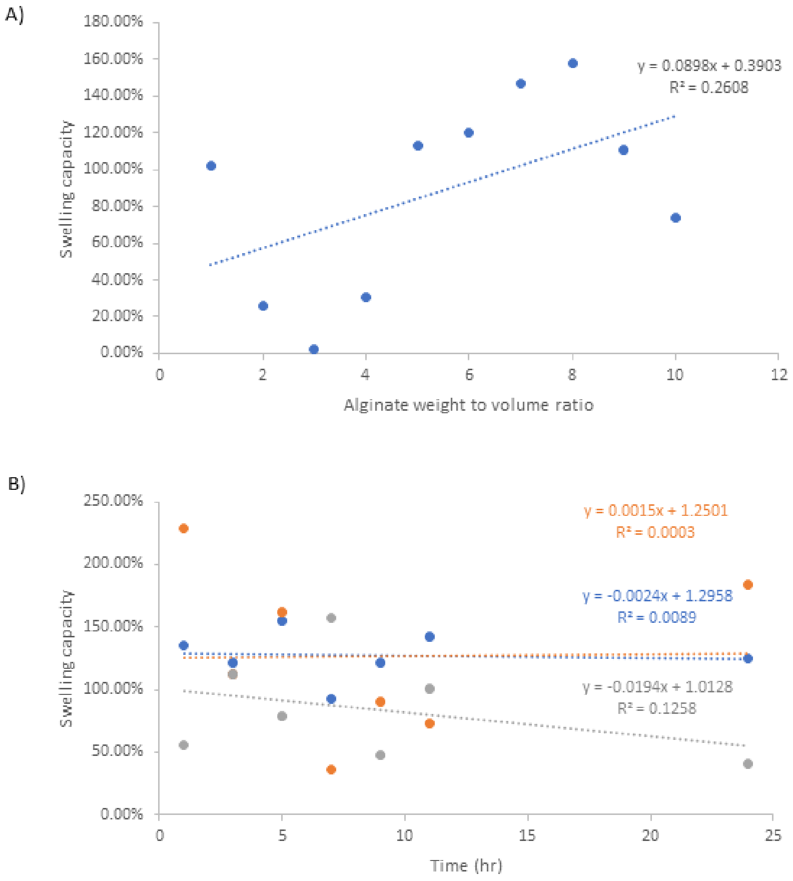

Figure S3 (**A**) Standard deviation for the swelling capacity of NA samples, (**B**) Standard deviation for the swelling capacity of NAA samples.

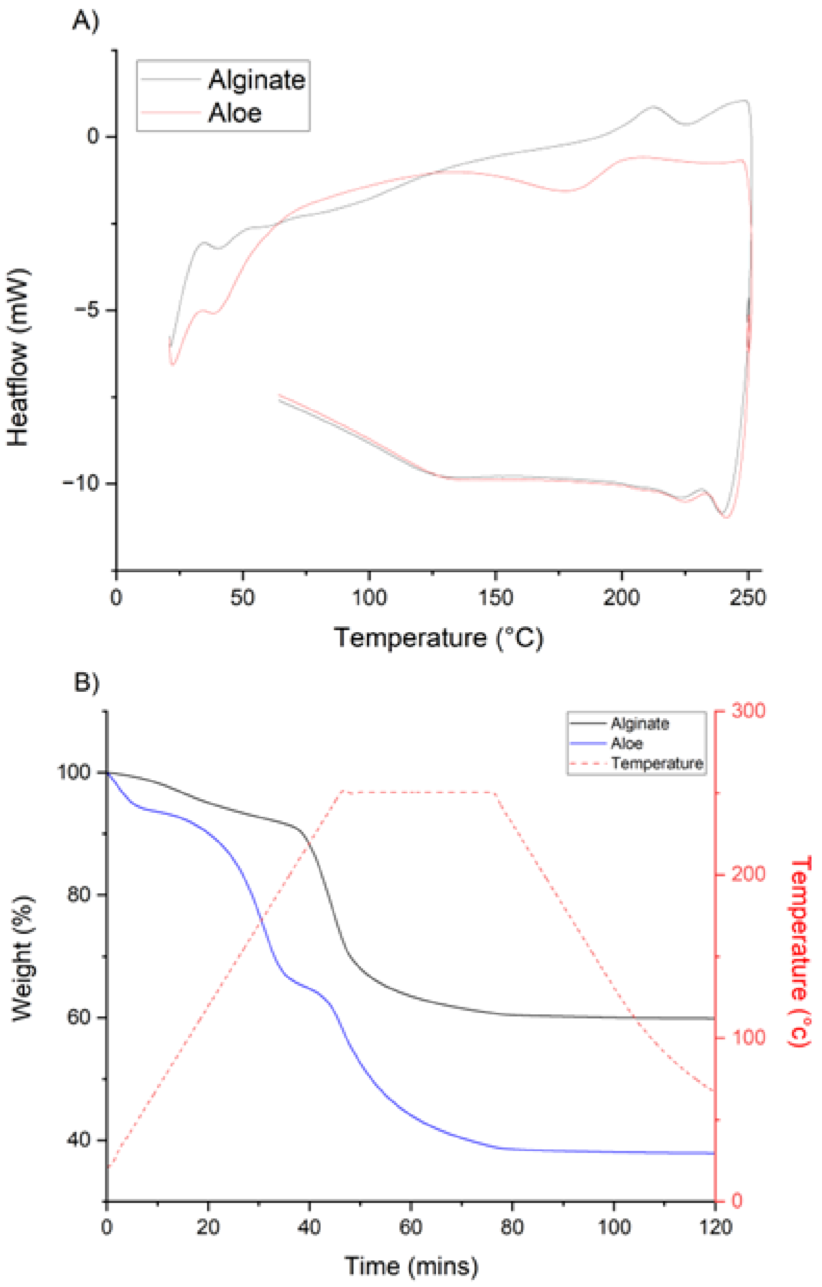

Figure S4 (**A**) Graph detailing the heat flow changes of aloe and sodium alginate as a function of temperature. (**B**) Graph detailing the weight changes of aloe and sodium alginate as a function of temperature.
